# The role of informational support from women’s social networks on antenatal care initiation: qualitative evidence from pregnant women in Uganda

**DOI:** 10.1186/s12884-022-05030-1

**Published:** 2022-09-16

**Authors:** Alison B. Comfort, Alison M. El Ayadi, Carol S. Camlin, Alexander C. Tsai, Hadija Nalubwama, Josaphat Byamugisha, Dilys M. Walker, James Moody, Tatyana Roberts, Umar Senoga, Paul J. Krezanoski, Cynthia C. Harper

**Affiliations:** 1 Bixby Center for Global Reproductive Health, Department of Obstetrics, Gynecology and Reproductive Sciences, University of California San Francisco, 550 16th Street, San Francisco, CA 94143 USA; 2 Bixby Center for Global Reproductive Health, Department of Obstetrics, Gynecology and Reproductive Sciences, University of California San Francisco, 1330 Broadway, Suite 1100, Oakland, CA 94612 USA; 3 Center for Global Health and Mongan Institute, Massachusetts General Hospital, Harvard Medical School, 125 Nashua Street, Suite 722, Boston, MA 02114 USA; 4 College of Health Sciences, School of Medicine, Makerere University, PO Box 7072, Kampala, Uganda; 5 Duke University, 268 Soc/Psych Building, Durham, NC 27708-0088 USA; 6 University of California San Francisco, Zuckerberg San Francisco General Hospital, 1001 Potrero Ave, San Francisco, CA 94110 USA

**Keywords:** Antenatal care, Uganda, Pregnancy, Social networks, Social support, Informational support

## Abstract

**Background:**

Early and appropriate use of antenatal care services is critical for reducing maternal and neonatal mortality and morbidity. Yet most women in sub-Saharan Africa, including Uganda, do not seek antenatal care until later during pregnancy. This qualitative study explored pregnant women’s reliance on social ties for information about initiation of antenatal care.

**Methods:**

We conducted semi-structured, in-depth interviews with 30 pregnant women seeking their first antenatal care visit at Kawempe Referral Hospital in Kampala, Uganda. Recruitment was done purposively to obtain variation by parity and whether women currently had a male partner. Study recruitment occurred from August 25^th^ 2020 – October 26^th^, 2020. We used thematic analysis following a two-stage coding process, with both deductive and inductive codes. Deductive codes followed the key domains of social network and social support theory.

**Results:**

We found that the most important source of information about antenatal care initiation was these women’s mothers. Other sources included their mothers-in-law, female elders including grandmothers, and male partners. Sisters and female friends were less influential information sources about antenatal initiation. One of the primary reasons for relying on their own mothers, mothers-in-law, and elder women was due to these women’s lived experience with pregnancy and childbirth. Trust in the relationship was also an important factor. Some pregnant women were less likely to rely on their sisters or female friends, either due to lack of trust or these women’s lack of experience with pregnancy and childbirth. The advice that pregnant women received from their mothers and others on the ideal timing for antenatal care initiation varied significantly, including examples of misinformation about when to initiate antenatal care. Pregnant women seemed less likely to delay care when more than one social tie encouraged early antenatal care.

**Conclusions:**

Educating women’s social networks, especially their mothers, mothers-in-law, and community elders, about the importance of early antenatal care initiation is a promising avenue for encouraging pregnant women to seek care earlier in pregnancy.

## Background

Maternal and neonatal mortality and morbidity remain high in sub-Saharan Africa, including in Uganda [1–4]. One critical strategy to reduce maternal and neonatal mortality and morbidity is to ensure early and appropriate use of antenatal care (ANC) services [5, 6]. Early and frequent ANC visits during pregnancy provide opportunities for screening, treatment, and prevention of complications while also offering education and support to ensure healthy pregnancies [7]. The World Health Organization’s 2016 guidelines recommend that pregnant women seek antenatal care as early as possible (within the first trimester) and have at least eight visits during pregnancy [6]. While almost all pregnant women in Uganda (97.5%) seek ANC services during pregnancy, most initiate care much later than recommended and few receive the recommended number of visits. Only 30% of pregnant women initiate ANC in the first trimester (< 4 months pregnant), half initiate care at 4–5 months pregnant, and 20% at 6 months or later. Only about 60% of Ugandan women have 4 or more ANC visits during their pregnancy and hardly any (2%) have 8 or more visits [4].

Our study focused specifically on timing of ANC initiation during pregnancy, since early initiation is a critical first step towards ensuring women receive continued ANC throughout pregnancy. Factors affecting timing of ANC initiation span several domains across multiple levels, as highlighted in recent systematic reviews and studies [8–11]. Early ANC initiation is more likely among women who are older, more educated and/or have a partner with more education, are employed, more economically empowered, married, living in urban areas, and/or those with greater exposure to mass media [8, 12–17]. Structural and economic barriers to early ANC initiation include long distance to facility, long wait times, and high indirect costs of care [8, 9, 12, 14, 16, 18–21]. Provider-related barriers are tied to poor quality of care, including mistreatment and lack of respectful care, shortages of medical supplies, and lack of individualized care [9, 19, 20, 22, 23].

Another critical domain affecting ANC initiation relates to women’s attitudes, beliefs, and subjective social norms about early ANC initiation [8, 9]. Some studies have shown that women believe early ANC initiation is only necessary for women with pregnancy complications [9, 12, 21], while others have shown that women may not know about timing of first ANC visit [14, 24]. Others have found that multi-parous women do not prioritize early ANC because of their prior experience with pregnancy [12, 15, 22], though women who experienced complications in prior pregnancies tend to initiate ANC early in subsequent pregnancies [12]. Several studies have highlighted women’s concerns about revealing or disclosing their pregnancy too early, due to cultural beliefs or fears of stigmatization [9, 20, 22]. Based on anecdotal knowledge of Uganda, most cultures hold it that a woman should not disclose her pregnancy early or even until delivery due to cultural beliefs that the pregnant woman can be bewitched or harmed by others outside her close social circle. While studies from other African countries find an association between late ANC initiation and desires to hide pregnancy [20, 22, 25, 26], there remains limited research specific to Uganda linking ANC initiation and hiding pregnancies despite this cultural practice. In many contexts in sub-Saharan Africa, care-seeking during pregnancy with traditional providers is normative compared to seeking care through the formal health sector [9, 22]. Despite the evidence showing the importance of women’s attitudes, beliefs and subjective social norms around timing of ANC initiation, there is very little research on who women rely on for information about ANC initiation, whether these individuals are close familial ties or weaker ties with non-familial relationships, and how this influences their care-seeking behavior.

### Theoretical framework

Our study examined who pregnant women relied on for informational support about ANC initiation. We used social network theory to examine how the patterns of relationships that connect individuals to each other influence women’s decision to seek ANC [27, 28]. These relationships may include social ties based on kinship, friendship, neighborhood, organizational affiliation and other groups [27, 29]. Social network theory suggests that health behaviors are, in part, driven by individuals’ networks because of the resources that flow through these networks. Network resources may be provided through informational, instrumental, emotional, and/or appraisal support and can affect access to opportunities and/or generate contraints on behavior [27, 30, 31]. In this study, we focused on informational support (among the different types of social support as set forth by Cohen & Wills [1985]) given the paucity of evidence around its role in influencing ANC initiation. Informational support can play two roles: first, it can represent pure information-sharing about the benefits of early ANC initiation. Second, it can be a way of conveying both descriptive norms (the perception of what most people do) and injunctive norms (the perception of what most people approve or disapprove of) [32].

While there exists a broader literature on the importance of social support in general on early ANC initiation [9], there is limited research examining who provides informational support and whether it varies by social ties. An ethnographic study among low-income, African American women in the United States found that women relied on their mothers, aunts and grandmothers for information about pregnancy [33]. A qualitative study among rural women in Mexico found that women were influenced by injuctive norms conveyed by their husbands, female family members and friends on the importance of seeking ANC during pregnancy, though information about timing of ANC initiation was not assessed [34]. Two recent systematic reviews identified very few studies in sub-Saharan Africa that looked at which social ties provided informational support about ANC initiation [8, 9]. One study in Mozambique found that male partners discouraged early ANC except if the woman was experiencing abdominal pain, while matrons and neighbors served as important decision-makers to encourage ANC seeking [23]. In constrast, in Gambia, both male partners and traditional birth attendants tended to be the ones to encourage prompt ANC initiation [35].

To fill a gap in our understanding on how social networks affect ANC initiation via informational support, we examined who pregnant women in Uganda relied on for information about timing of ANC initiation and how this information varied by social tie. Drawing on the strength of weak ties theory [36], we assessed why women relied on different ties. They may rely on strong ties (i.e., familial/social ties with greater contact, intimacy, emotional intensity and reciprocity) because they are trusted, represent important sources of social support, even though they may also exert more pressure to conform with social norms [36, 37]. Alternatively, women may rely on weak ties (e.g. neighbor, health worker, non-kin social tie) because these social ties tend to be bridges to other social groups with novel or different information [36]. Weak ties may also be preferred if they are similar based on attritubes or similar experiences or circumstances [38].

By better understanding what information women receive about ANC initiation and from whom, we can design more effective interventions that engage women’s social networks to increase the proportion of women who initiate ANC early in pregnancy. Thus, we conducted exploratory research among pregnant women in Uganda to better understand who they relied on for information about ANC initiation and what type of information they received from these ties.

## Methods

We conducted semi-structured, in-depth interviews with 30 pregnant women seeking their first ANC visit at Kawempe National Referral Hospital (known as Kawempe Hospital) in Kampala, Uganda.

### Study setting and study participants

Study participants were recruited from Kawempe Hospital, the public referral hospital for the Kampala metropolitan area, with approximately 170 beds, and 30,000 annual births. It serves as a teaching hospital for Makerere University College of Health Sciences. Kawempe Hospital has an outpatient clinic where pregnant women receive ANC.

Eligibility criteria for the study included women who were currently pregnant, ages 18 years or older, and were visiting Kawempe Hospital for their first ANC visit. We recruited women at their first visit to reduce recall bias about events earlier in their pregnancy. A total of 30 women were included in the study; this sample size was reached based on thematic saturation. Recruitment was done purposively to obtain variation by whether women currently had a stable male partner. Half of women in the sample were in partnership (i.e., married, engaged to be married, or in non-marital partnership) and half identified as having no current partner nor living with a partner.

Women in partnership were recruited along with their male partner for the purposes of a separate dyadic analysis. Only those whose partner was in attendance at the first visit were recruited. This adaptation in recruitment was necessary due to COVID-19 restrictions, because we could not ask the partner to make a separate trip to Kawempe Hospital for an in-person interview.

Study recruitment started once in-person ANC visits (which were disallowed for a time due to the COVID-19 pandemic) resumed at Kawempe Hospital. They occurred from August 25^th^, 2020 to October 26^th^, 2020.

### Participant recruitment and data collection

Women were recruited prior to entering the examination room for their ANC visit. The research team administered the study screening questions (currently pregnant, >  = 18 years old, first ANC visit, partnership status, and whether partner was present at time of visit). If a study participant screened eligible, she was asked to meet with the research team after her visit. For the in-depth interview, the research team invited the study participant to a private room where they first administered a COVID-19 screening process (temperature, symptom screening, and exposure to known cases). The research team then explained study procedures, risks, and benefits. All study participants provided written confirmation of informed consent, including consent for medical record reviews of gestational age at that ANC visit. Interviews were conducted by trained qualitative interviewers in Luganda, the local language. Interviews lasted, on average, 70–90 min. The interviewers audio-recorded the interviews and took notes while interviewing. After the interviews, the research team transcribed the interviews verbatim then translated them to English for analysis.

Before the in-depth interview, the interviewer administered a brief questionnaire on participant socio-demographics, parity, care-seeking during prior pregnancies, self-reported gestational age, and quality of relationship with partner (if applicable). The semi-structured interview guide was informed by key domains based on the Berkman et al. conceptual model of social networks and health [27] and the different domains of social support from Cohen and Wills [31]. These domains included social supports (informational, emotional, instrumental and appraisal support), social norms, and attitudes and beliefs (in this case, about ANC initiation during pregnancy). Study participants were asked who provided the informational support and the domains of informational support salient to the study’s research questions (e.g., timing of ANC visit, benefits of ANC, etc.). Once interviews were completed, the interviewer reviewed the woman’s ANC card to record her gestational age at this first ANC visit.

### Data analysis

We used semi-structured in-depth interviews to collect detailed qualitative data on particular themes, concepts and ideas, while also adapting interview questions with probes and follow-up questions to permit exploration of unexpected topics as they arose during the discussions with study participants [39]. Once an initial set of 10 interviews were conducted, we reviewed the transcripts, adapted questions to further explore certain themes, and added some probes to obtain more details about certain topics. We also adjusted our purposive sampling to ensure variation in parity. Once data collection was completed and transcripts were transcribed and translated, we uploaded data to ATLAS.ti for analysis.

We used thematic analysis following a two-stage coding process [40, 41]. In the first stage, AC reviewed a set of transcripts to identify key concepts as potential codes and generated a codebook with code definitions. Coding included both deductive and inductive codes. Deductive codes followed the key domains from Berkman et al.’s theoretical framework of social networks and health behavior and Cohen and Wills’ domains of social support [27, 31]. Inductive codes were later added when other topics were emergent in the data from inductive, line-by-line coding. For example, codes for elderly women in the community and neighbors were added, as well as the internet. AC, HN, and TR met to review the codes in the codebook and clarify code definitions. HN and TR independently coded the same 4 transcripts. To ensure consistency across coders, the coders met to compare coding, further refine and adjust coding definitions and add additional codes as needed. In the second stage, HN and TR independently coded the transcripts, and met consistently together with AC to discuss coding questions and resolve any discrepancies in the ways codes were applied. Once all transcripts were coded, AC focused specifically on data coded under informational support. Among these data, AC employed focused coding to identify the main types of informational support based on inductive codes. Once data coding and analysis were completed, the authors agreed on the final themes.

## Results

### Descriptive characteristics of sample

Participants were largely 25 years old or younger and most were nulliparous, although 2 individuals had 6 and 8 prior births (Table 1). Consistent with our purposive sampling strategy, half of study participants were in partnership and half had no stable male partner. More than half of participants had reached secondary school. Data from the participants’ ANC health records showed that only 6 participants were in their first trimester (12 weeks gestation or less) at this first ANC visit. Half of study participants (*n* = 14) were in their second trimester of pregnancy (13–27 weeks), and 10 were in their third trimester (28 weeks or more).

**Table 1:** Descriptive characteristics of study sample (*n* = 30)

	*n*	%
**Demographics**		
Age		
18–19	8	27
20–25	16	53
26–30	2	7
31 +	4	13
Parity		
0	19	63
1	9	30
2–4	0	0
5 +	2	7
Relationship status		
Married or living with partner	15	50
No partner	15	50
Highest schooling attained		
Primary	7	24
Secondary	17	56
Tertiary or more	6	20
Mean household size (SD)	4.1	(4.14)
**ANC seeking**		
Gestational age at first ANC visit *(from ANC card)*		
First trimester (< = 12 weeks)		
7 weeks	1	3
10 weeks	1	3
12 weeks	4	13
Second trimester (13- 27 weeks)		
16 weeks	5	17
17 weeks	1	3
20 weeks	6	20
24 weeks	1	3
26 weeks	1	3
Third trimester (28 + weeks)		
28 weeks	6	20
30 weeks	4	13

### Women’s beliefs about benefits of early ANC initiation

Women shared many reasons that they believed it was beneficial to initiate ANC early in pregnancy. These reasons included: to confirm the pregnancy, receive medications and immunizations (e.g., folic acid, anti-malarials, tetanus shot), and test for HIV and sexually transmitted infections (STIs). Several women mentioned the benefit of learning if the baby was well-positioned, ruling out ectopic pregnancies, monitoring the baby’s heartbeat and health, and receiving the antenatal card.

When asked what women should know about seeking ANC once they confirm they are pregnant, one study participant responded:

“[…] I would tell women that seeking antenatal care has no harm and the earlier you go to the hospital, the better. It is not good to choose to go to the hospital at 6 months or once just to get the antenatal card. We don’t know what happens to these babies during pregnancy unless you go to the hospital and the health provider tells you what is happening.”

-27 years old, primiparous, 10 weeks pregnant

Another woman (28 weeks pregnant at her first ANC) said she believed: “most complications are due to failure to begin antenatal care early.” Another woman said she wished she had attended ANC earlier to:

“Learn the baby’s condition and what is missing in the uterus to help it grow. They have told us that there are tablets they give to us at the beginning of pregnancy to nourish where the baby will be attached in the uterus, but I didn’t get them.”

-24 years old, nulliparous, 30 weeks pregnant

However, a few women believed that early ANC initiation was not necessary unless a woman was experiencing complications. Indeed, a few women said the only reason they had initiated ANC early in this pregnancy was because of physical symptoms that concerned them (e.g., stomach pain, headaches, or suspected infection). One study participant (28 weeks pregnant at first ANC) said she had been hospitalized for complications: “When they discharged me, the doctor wrote on my card, ‘start antenatal.’”

Most study participants started ANC in their second trimester or later. Yet, when asked about their beliefs on the ideal timing to start ANC, many said they believed it should happen as soon as a woman finds out she is pregnant. Several women said women should come when they are 2–3 months pregnant. Others said the first ANC should happen at 4–5 months pregnant. Several women also rationalized that early ANC initiation would allow them to meet the expected 8 visits during the pregnancy. However, there were also examples of women who thought ANC could start at any time during pregnancy. One woman said:

“Women come at 4 or even 6 months. So, it depends on what the mother prefers. Some mothers prefer 2, 3 or even 7 months.”

-18 years old, nulliparous, 28 weeks pregnant

One study participant, who had 8 prior births, expressed her reticence in starting ANC early:

“Imagine coming every month!”

-33 years old, multi-parous, 16 weeks pregnant

Indeed, several study participants did not believe that early ANC initiation was necessary. One woman replied that she would not want to come early:

“Personally, I would wait for as long as I am not in pain.”

-22 years old, multi-parous, 28 weeks pregnant

There was substantial variation in study participants’ as to the ideal timing for the first ANC visit and the perceived benefits of initiating ANC early. Thus, we further explored who was providing these women with informational support about early ANC initiation.

### Sources of informational support about timing of ANC initiation

We found that study participants relied on a number of different social ties for information about ANC initiation. Most frequently, pregnant women relied on their own mothers. Many also relied on their mothers-in-law (if they were partnered) and on elder women in the community, including their grandmothers. While male partners also played a role in providing information and advice about the timing of ANC visit, they were more involved in decision-making around where to seek ANC. In contrast, pregnant women were less likely to rely on female friends, sisters, and aunts though some did turn to them for informational support. Health care workers were also mentioned as a source of information about the benefits of early ANC.

#### Mothers

One of the most common social ties providing information about early ANC initiation were mothers. Many study participants reported that they were encouraged or even told by their mother to seek ANC. One woman said that, when she experienced bleeding during her pregnancy, it was her mother who advised her to go to the hospital.

There was variation in terms of advice from mothers about the ideal timing of the first ANC visit. One study participant, who revealed her partner was against her seeking ANC early in pregnancy, said her mother thought she should have sought ANC in the first trimester.

“[My mother] told me, “If the pregnancy is 4 months, you are already late for antenatal care. You should start.” I didn’t tell her that my partner had refused the idea, I just told her that I haven’t gone because I feel fine. Besides, I thought it’s only those with complications that go for antenatal care. She advised me to go for antenatal care immediately.”

- 24 years old, nulliparous, 20 weeks pregnant

Another study participant received advice from both her mother and female friends to seek ANC at 4 months:

“Mother told me to begin at 4 months and even the women I talked to told me to begin at 4 months. So, I decided that the best time was at 4 months.”

- 19 years old, nulliparous, 17 weeks pregnant

In contrast, another study participant’s mother did not believe there was a specific time to start ANC. The woman explained…

“When I got pregnant, I didn’t know when to start antenatal, I just asked my mother because she has just given birth from [this hospital] […] She told me, ‘anytime.’”

- 23 years old, nulliparous, 12 weeks pregnant

There are several reasons why pregnant women seem to rely on their own mothers for advice about timing of ANC initiation. First, several participants shared that it was culturally expected for mothers to advise their daughters on the importance of ANC and its timing. Another common reason was tied to daughters’ trust in their mothers. As one participant shared: “Mothers can’t give bad advice because she wishes me the best.” Several participants mentioned that their mothers would be honest with them. One participant said that her concerns about revealing her pregnancy to her mother were overridden by the sense of trust:

“Personally, on my first pregnancy, I was worried that my mother would beat me or do something, but I trusted both [my parents]. They know me and I trust them. In fact, they didn’t beat me, but instead treated me well. So, why run to someone else who will gossip about me?”

- 22 years old, primiparous, 28 weeks pregnant

Another reason related to mothers’ own experience with pregnancy and childbirth themselves, which led women to respect their mothers’ views and advice.

“It’s because I trust her [mother] and she has a lot of experience…. Because it’s [my mother and grandmother] who have helped my sisters give birth well.”

- 20 years old, nulliparous, 12 weeks pregnant

#### Mothers-in-law

While pregnant women most frequently mentioned their mothers as sources of informational support about ANC visits, there were many examples of women also relying on their mother-in-law. When asked who was advising her on ANC, one study participant said:

“I asked mother, mother-in-law, and my neighbors. They all know that I have come here for antenatal care……My mother-in-law had advised [me] to begin at 3 months.”

- 19 years old, nulliparous, 17 weeks pregnant

Similar to why women chose to rely on their own mothers for advice about ANC, some chose to turn to their mothers-in-law because of their experience with pregnancy and childbirth. As one participant shared:

“They [mothers-in-law] know how it feels to be pregnant so she can advise you.”

- 31 years old, nulliparous, 28 weeks pregnant

However, there was much more variation between study participants as to whether they wanted to turn to their mother-in-law for information about ANC and its timing. In some examples, there was significant trust, including because women may be living with their in-laws as is traditional when women marry into their partner’s family. When asked who she turned to for information about ANC and pregnancy, one study participant said:

“My mother-in-law because she is the one whom I stay with and whenever I feel any discomfort, I tell her about how I feel, and she advises me on what to do.”

- 19 years old, nulliparous, 17 weeks pregnant

In contrast, several study participants said there were tensions in their relationship with their mother-in-law. One study participant said that she did not feel emotionally close to her mother-in-law even though she would speak to her on the phone. Other study participants relayed their reluctance to rely on the mother-in-law for advice about ANC because:

“There are mothers-in-law who can’t let you be in peace.”

- 31 years old, nulliparous, 28 weeks pregnant

“Some mothers-in-law are not easy, but some are. Most of them are hard; you cannot tell your mother-in-law everything. In fact, your friend might even be better than your mother-in-law.”

- 20 years old, multiparous, 16 weeks pregnant

Another reason why women may be more likely to turn to their own mothers for advice about ANC initiation may be cultural. As one study participant explained, she was more likely to rely on her own mother for information *during* pregnancy and her mother-in-law for information *after* delivery.

“I would ask her [my mother] what to do and not do during pregnancy. *(After birth)* I would ask mother-in-law [about issues related to the baby] […] [My mother-in-law] might know how to hold the baby and how to deal with the umbilical cord. If I ask my mother, she will tell me about things they do in my tribe.”

- 24 years old, nulliparous, 20 weeks pregnant

This contrast suggests the important role that women’s own mothers play during pregnancy, as compared to the role of the partners’ family after birth with the baby.

#### Male partners

In many instances, male partners also played an important role in providing advice and information about ANC and its timing, particularly among women in partnership. Several study participants mentioned it was their partner/husband who had encouraged them to come to the hospital for ANC. Even among women who believed ANC visits were only for women experiencing complications, it was the male partner who encouraged them to seek ANC. One study participant shared:

“I had no complication and I also heard that antenatal care is a waste of time. I heard that they [health workers] only tell us to come back to the hospital for nothing. When I told him [husband] about all that, he told me to come and see because it might not be a waste of money.”

- 22 years old, nulliparous, 20 weeks pregnant

Similar to the role of mothers, partners also advised on the timing of ANC visits. When asked who had encouraged her to come to the ANC visit, one woman said:

“It was my husband. He told me, ‘you are delaying the antenatal visit, and you will hurt the child!’ but I kept on telling him that I will go. I wasn’t feeling any discomfort, so I thought [I] was fine. Then, this friend of mine kept on saying, ‘days are running out, you are going to deliver without receiving antenatal care. You don’t know how the child is. You don’t know your health status. You have to go to the hospital!’”

- 28 years old, nulliparous, 28 weeks pregnant

Another woman named both her partner and mother as the ones who encouraged her seek ANC early:

“I didn’t want to come for antenatal before 6 months, but my mother and partner insisted that I should come since I had some complications in the beginning.”

- 22 years old, nulliparous, 20 weeks pregnant

As compared to mothers, partners were less frequently mentioned in providing advice about the timing of ANC visits as compared to helping decide where to seek care. Indeed, community expectations for the male partner centered more around decision-making about where to seek care and their involvement in accompanying the woman to the ANC visit. One woman shared her conversation with her partner about where to seek care:

“He suggested Kawaala Health Centre, but I told him that it is far and inconvenient to me in case I got a complication. So, he said that we’ll come here and see how it goes.”

- 22 years old, nulliparous, 20 weeks pregnant

However, there were also several examples where partners were not involved in providing advice about ANC, especially among women who considered themselves to be single but still had contact with their former partner. Several of these study participants said they received no social support from their formal partner and made the decision about when to seek ANC on their own. One study participant (30 weeks pregnant at first ANC; not in partnership) shared: “He doesn’t know about antenatal care” and another study participant (26 weeks pregnant) said: “I, who is pregnant, must be the one to decide [when to seek ANC] […] because he doesn’t know when.” Another study participant specifically did not seek informational support from her former male partner due to concerns about domestic violence. In addition, there are also examples where male partners discouraged ANC initiation whereas the mother was encouraging it.

#### Elder women in the community including grandmothers

Elder women in the community, including grandmothers, were another frequently mentioned source of information about ANC and its timing. Women who mentioned relying on their grandmothers were more likely to turn to them for information about pregnancy in general and how to behave during pregnancy, with only a few examples of grandmothers conveying the importance of seeking ANC.

Many women also mentioned elder women in the community who were not related to them. These women were not community leaders but rather respected community members by virtue of their age and experience. The advice from elders focused more on the importance of seeking ANC rather than specifics about timing of ANC initiation.

“There is an elderly woman who is also a neighbor, she told me '[…] this is [your] first child […] you need to go to the hospital and get some medication. You will never know what will happen.' It was her advice that made me come here for antenatal care.”

- 31 years old, nulliparous, 17 weeks pregnant

In another similar example (where the study participant was 26 weeks pregnant at her first ANC), her elder neighbor (whom she considered a “grandmother”) encouraged her to seek ANC as soon as possible. In certain cases, women actively sought the advice of elders in the community. In other cases, they received unsolicited advice.

“You know how those older women can notice things easily, and after noticing they start telling you what to do […] I didn’t choose her [an old woman who is her neighbor]; she just called and talked to me as an elder […] When that lady came to me, I thought I could use her advice […] She told me to come to the hospital since it’s my first pregnancy.”

-31 years old, nulliparous, 28 weeks pregnant

Similar to the reasons for relying on mothers and mothers-in-law, the main reason for turning to elder women or choosing to follow unsolicited advice from these elder women was due to these women’s own prior experience with pregnancy and childbirth. In many instances, study participants specifically cited these elders’ experience with pregnancy and childbirth as reasons for turning to them. One study participant explained who she would seek informational support from:

“Someone who knows about pregnancy…an older person…They have more experience compared to the young ones.”

-31 years old, nulliparous, 28 weeks pregnant

In the case of grandmothers, it is the familial ties and trust that may also explain women’s reliance on them. A 30-week pregnant women said she would have turned to her grandmother during this pregnancy because she was the one to provide her with advice during her prior pregnancy, but her grandmother had recently passed away.

#### Sisters, female friends, and aunts

Sisters, female friends, and aunts were also mentioned as sources of information about ANC and the timing of ANC initiation, although much less frequently than mothers and mothers-in-law. There were several examples of women turning to sisters, friends and aunties for information about ANC. One study participant (12 weeks pregnant at her first ANC) said it was her friend who had encouraged her to seek ANC immediately.

One study participant, who was living outside of Uganda when she became pregnant, was encouraged by both her sister and her aunt to seek ANC as soon as she returned to Uganda. When asked if her sister and aunt had influenced her decision to seek ANC at 7 months pregnant, the woman shared:

“Yes, because it’s what they want. They want to make sure I get enough treatment by the time I give birth. So, everyone supports me to start antenatal.”

-24 years old, nulliparous, 30 weeks pregnant

Among the women who did rely on their sisters, friends and aunts, they appeared to do so because those individuals had also had experience with pregnancy and childbirth. In some cases, study participants were currently living with their aunts.

One participant explained why she turned to her friend:

“I would ask my friend who is a new mother but there are times I feel that she may not know, so I go back to asking Google [….] Because [my friend] has just given birth and has gone through it all well. She has fresh information.”

-22 years old, nulliparous, 20 weeks pregnant

Another participant shared why she relied on her sisters:

“I am the last born in our home so, my sisters have all the experience….[On] pregnancy, giving birth and taking care of the baby.”

-25 years old, nulliparous, 7 weeks pregnant

However, there were also many instances where women specifically chose not to rely on friends or sisters for information about ANC. One main reason was because their friend or sister was young and did not have any experience with pregnancy. As one study participant said: “[My friend] is the same age as me so there is nothing much she knows.”

Trust in their friends and sisters also seemed to be a discerning factor for whether women chose to rely on them for informational support. There was variation in the sample regarding the level of trust in their friends and sisters. Several study participants said they did not trust their friends; some even mentioned being concerned that their friends might want to harm them or the baby through witchcraft. One study participant explained:

“Sometimes you might disclose to a friend thinking that she is your friend but when she is your co-wife! Alternatively, she could give you a drink which would make you lose your pregnancy.”

-19 years old, multiparous, 26 weeks pregnant

In the case of aunts, some relied on their aunts because they lived with them and/or viewed them similar to a parent. In other cases, some women chose not to rely on them for fear of their reaction about the pregnancy.

“I have always gotten pregnant while still staying with my aunt so, I believe sometimes we fear telling them about it because we are afraid of them, so we confide in our friends.”

-38 years old, multiparous, 12 weeks pregnant

#### Health providers

In addition to social ties, study participants also mentioned that health providers served as a source of information about ANC and its timing. Several study participants said they had learned from health providers about the importance of early ANC seeking. As a result, many of the study participants confirmed that they thought it was important to start ANC at 2–3 months pregnant or as soon as they missed their period. One woman explained:

“The health care workers teach that it is good to attend antenatal care early […] The health workers have advised [to come at] one month and that’s what I want to do.”

-20 years old, multi-parous, 20 weeks pregnant

One of the most frequently cited reasons for relying on health providers for information about ANC and timing of initiation relates to their training and expertise. As one 24-year-old nulliparous said, she relied on the doctor because: “he knows best and provides the final answer.” Health workers were viewed as trusted sources of information because of the depth of their knowledge. Several participants shared similar sentiments that health workers: “know best”, “know more”, and “are the ones who know everything.” Another person provided a religious reference:

“We are all supposed to come for antenatal care because the health care providers are gifted by God to see what needs to be seen.”

-33 years old, multiparous, 16 weeks pregnant

## Discussion

This qualitative study reveals that pregnant women’s social networks play an important role during pregnancy in providing informational support related to timing of ANC initiation. Overall, mothers seem to be the most influential information source about the benefits of ANC and the timing of the first visit. Other important social ties for information about ANC initiation include the mother-in-law (for women in partnership) and female elders in the community, including grandmothers. In some cases, sisters, female friends and aunts also played a role in providing informational support about ANC, but not all women turned to these social ties. While the male partner also provides information and advice about ANC initiation, they are much more likely to be involved in decision-making about where to seek ANC and accompanying the woman to the visit.

Together, these findings are consistent with other research showing that social ties can be important sources of information about ANC seeking [8, 9, 23, 35]. Our results are similar to other studies showing that pregnant women may rely on male partners [23, 34, 35], female friends [34], female family members [34], including mothers-in-law [42], and neighbors including elders [23]. However, we also found that certain ties, such as male partners, may discourage ANC, similar to findings in Mozambique with partners wanting the pregnant woman to delay care-seeking [23]. We also identified some tensions in relying on mothers-in-law, similar to findings in Nepal where early ANC seeking was less likely because mothers-in-law discouraged it [42]. While the internet was not a commonly cited source of information about ANC, a few study participants did mention relying on internet searches to confirm information from social ties.

Lastly, health workers also represented important informational sources about the benefits of ANC including early ANC initiation. These findings are somewhat similar to evidence in Mozambique and Gambia with women relying on health providers, traditional birth attendants, matrons and community health workers as informational sources about ANC initiation [23, 35]. They are consistent with evidence from Madagascar where individuals are just as likely to rely on social ties versus health providers for reproductive health advice and information [43, 44]. Yet, these findings may reflect what pregnant women learned during their ANC visit and may not represent the key information sources leading them to first seek ANC.

Our findings directly contribute to the strength of weak ties theory by Granovetter (1973) and most recently confirmed with research by Small (2017). Pregnant women in our sample relied not only on strong, close familial ties (e.g., mothers, mothers-in-law) but also on weaker ties (e.g. non-familial elder women in the community). Their mothers, and in some cases mothers-in-law, represent familial ties characterized by greater contact, intimacy, emotional intensity and reciprocity [36,37] and trust was an important reason for relying on them. Women turned to their own mothers, even before their partner, because they knew their mothers wanted the best for them. Women were judicious about relying on their mothers-in-law depending on tensions in these relationships and the pressures to conform to social norms from the male partner’s family.

Pregnant women also relied on weak ties (non-familial ties with less intimacy, frequency of contact, and emotional intensity), consistent with findings from Small et al. [38]. These ties included non-familial elder women in the community and health workers. They tended to rely on elder women because of their own past experience with pregnancy and their culturally important community role. They chose to rely on health workers due to their expertise through training and education. A key overarching theme for relying on both strong and weak ties was due to these individuals’ lived experience with pregnancy and childbirth. These findings are consistent with the theory that women may turn to social ties (including weak ones) because of attribute and situational similarities [38]. This preference also explains why some pregnant women did not rely on female friends or sisters if they were young or had not yet been pregnant.

Pregnant women themselves held a range of beliefs about the importance of early ANC initiation. Some believed ANC was only necessary for women with complications, while others saw the benefit of attending ANC early to confirm the pregnancy, receive medications and immunizations, test for infections, and monitor their own and their baby’s health. Yet, there was substantial variation in when they thought they should initiate their first visit: ranging from as soon as she confirms her pregnancy, at 2–3 months, 4–5 months, or anytime. While social ties encouraged ANC seeking, their advice on timing of ANC varied and some provided misinformation ideal timing. Among the mothers who were mentioned, some suggested seeking ANC immediately, while others said it was ideal at 4 months, and others believed it could happen anytime. When pregnant women received information from more than one social tie, especially if the information was consistent, seemed to lead women to avoid delaying ANC. In cases where advice conflicted (such as when the mother encouraged prompt ANC while the partner did not support it), it appeared that pregnant women followed the advice of those with lived experience or expertise with pregnancy. However, there were many examples where social ties did not encourage early ANC initiation which may be an important contributor to delayed ANC seeking.

This study has certain limitations. It was conducted during the COVID-19 pandemic which limited recruitment of women in partnership to those attending ANC with their partner in person. Women who are in partnership but attend ANC without their partner may be different across key characteristics. Women in our sample tended to be in their first or second pregnancy and those with complications; few were multiparous. Multiparous women may further delay or forgo ANC because of prior experience with pregnancy and childbirth [12, 22]. Nulliparous women also tend to be younger, so our findings do not reflect older reproductive age women. The study was conducted in an urban context at a referral hospital with an ANC clinic. The findings may not reflect information sources important to rural women, such as traditional birth attendants. There were many examples (not reported) of women visiting traditional birth attendants to access traditional medicine for pregnancy. Given that recruitment occurred at a referral hospital, the sample does not reflect women who seek ANC from health workers at local health clinics. Thus, our sample may differ by socio-demographics and/or be more likely to include women with complications who prefer a referral hospital. This qualitative study does not provide causal evidence of the effect of informational support on timing of first ANC visit, and instead uncovers which ties are the most important information sources about ANC initiation. Additional data on tie strength would help us better understand how study participants conceived of the quality of their relationships. Because interviews were conducted after the first ANC visit, data about health providers likely represent what pregnant women learned during this first ANC visit and may not reflect whether health providers were key information sources about ANC initiation.

## Conclusions

Women’s social networks serve as an important source of information about the benefits of ANC and timing of its initiation. Our findings bring forth the important role of mothers, mothers-in-law, and elder women in the community as information sources about ANC initiation. They play an important role by virtue of being familial, trusted social ties. Non-familial elder women also served as important information sources due to their lived experience with pregnancy; similar results were true for sisters and friends who were older and had been previously pregnant. While male partners also served as information sources, they were more involved in decision-making about where to seek ANC. The advice on timing of ANC varied with some ties suggesting it was not necessary to seek ANC early in pregnancy. Pregnant women seemed less likely to delay ANC when more than one social tie encouraged prompt ANC. To ensure earlier ANC initiation, it is therefore critical to engage women’s social ties and educate them on the benefits of early ANC initiation so that they can support earlier ANC seeking.

## Data Availability

The datasets used and/or analyzed during the current study are available from the corresponding author on reasonable request.

## Abbreviation

ANC: Antenatal care
STIs: Sexually transmitted infections

## References

[1] UNICEF. "Neonatal mortality 2018" *UNICEF *December 2021. Accessed: July 25, 2022. Available from: https://data.unicef.org/topic/child-survival/neonatal-mortality/. Accessed: 25 July 2022.

[2] WHO, Unicef, UNFPA, The World Bank (2019). Trends in maternal mortality: 2000 to 2017.

[3] UN Inter-agency Group for Child Mortality Estimation. Levels and Trends in Child Mortality: Report 2021. New York, NY: UNICEF; 2021. https://www.who.int/publications/m/item/levels-and-trends-in-child-mortality-report-2021.

[4] Uganda Bureau of Statistics, ICF. Uganda demographic and health survey 2016. Kampala, Uganda and Rockville, Maryland, USA; 2018.

[5] Darmstadt GL, Bhutta ZA, Cousens S, Adam T, Walker N, de Bernis L (2005). Evidence-based, cost-effective interventions: how many newborn babies can we save?. Lancet.

[6] WHO. WHO Recommendations on antenatal care for a positive pregnancy experience. Geneva, Switzerland: World Health Organization; 2016. 28079998

[7] Lincetto O, Mothebesoane-Anoh S, Gomez P, Munjanja S. Opportunities for Africa's newborns. The partnership for maternal NaCH, editor. Geneva, Switzerland: WHO; 2012.

[8] Okedo-Alex IN, Akamike IC, Ezeanosike OB, Uneke CJ (2019). Determinants of antenatal care utilisation in sub-Saharan Africa: a systematic review. BMJ Open.

[9] Downe S, Finlayson K, Tuncalp O, Gulmezoglu AM (2019). Provision and uptake of routine antenatal services a qualitative evidence synthesis. Cochrane Database Syst Rev.

[10] Shitie A, Azene ZN (2021). Factors affecting the initiation and continuation of maternal health service utilization among women who delivered in the past one year in Enemay district, East Gojjam. Ethiopia Arch Public Health Archives belges de sante publique.

[11] Pervin J, Venkateswaran M, Nu UT, Rahman M, O'Donnell BF, Friberg IK (2021). Determinants of utilization of antenatal and delivery care at the community level in rural Bangladesh. PLoS ONE.

[12] Simkhada B, Teijlingen ER, Porter M, Simkhada P (2008). Factors affecting the utilization of antenatal care in developing countries: systematic review of the literature. J Adv Nurs.

[13] Sserwanja Q, Mutisya LM, Musaba MW (2022). Exposure to different types of mass media and timing of antenatal care initiation: insights from the 2016 Uganda demographic and health survey. BMC Womens Health.

[14] Turyasiima M, Tugume R, Openy A, Ahairwomugisha E, Opio R, Ntunguka M (2014). Determinants of first antenatal care visit by pregnant women at community-based education, research and service sites in Northern Uganda. East Afr Med J.

[15] Bbaale E (2011). Factors influencing timing and frequency of antenatal care in Uganda. Australas Med J.

[16] Atuhaire R, Atuhaire LK, Wamala R, Nansubuga E (2020). Interrelationships between early antenatal care, health facility delivery and early postnatal care among women in Uganda: a structural equation analysis. Glob Health Action.

[17] Sserwanja Q, Nabbuye R, Kawuki J (2022). Dimensions of women empowerment on access to antenatal care in Uganda: A further analysis of the Uganda demographic health survey 2016. Int J Health Plann Manage.

[18] Fagbamigbe AF, Idemudia ES (2015). Barriers to antenatal care use in Nigeria: evidences from non-users and implications for maternal health programming. BMC Pregnancy Childbirth.

[19] Mason L, Dellicour S, Ter Kuile F, Ouma P, Phillips-Howard P, Were F (2015). Barriers and facilitators to antenatal and delivery care in western Kenya: a qualitative study. BMC Pregnancy Childbirth.

[20] Pell C, Meñaca A, Were F, Afrah NA, Chatio S, Manda-Taylor L (2013). Factors affecting antenatal care attendance: Results from qualitative studies in Ghana, Kenya and Malawi. PLoS ONE.

[21] Kisuule I, Kaye DK, Najjuka F, Ssematimba SK, Arinda A, Nakitende G (2013). Timing and reasons for coming late for the first antenatal care visit by pregnant women at Mulago hospital. Kampala Uganda BMC Pregnancy Childbirth.

[22] Jinga N, Mongwenyana C, Moolla A, Malete G, Onoya D (2019). Reasons for late presentation for antenatal care, healthcare providers' perspective. BMC Health Serv Res.

[23] Munguambe K, Boene H, Vidler M, Bique C, Sawchuck D, Firoz T (2016). Barriers and facilitators to health care seeking behaviours in pregnancy in rural communities of southern Mozambique. Reprod Health.

[24] Kawungezi PC, AkiiBua D, Aleni C, Chitayi M, Niwaha A, Kazibwe A (2015). Attendance and utilization of antenatal care (ANC) services: multi-center study in upcountry areas of Uganda. Open J Prev Med.

[25] Kim KH, Choi JW, Oh J, Moon J, You S, Woo Y (2019). What are the barriers to antenatal care utilization in Rufisque District, Senegal?: a bottleneck analysis. J Korean Med Sci.

[26] Chimatiro CS, Hajison P, Chipeta E, Muula AS (2018). Understanding barriers preventing pregnant women from starting antenatal clinic in the first trimester of pregnancy in Ntcheu District-Malawi. Reprod Health.

[27] Berkman LF, Glass T, Brissette I, Seeman TE (2000). From social integration to health: Durkheim in the new millennium. Soc Sci Med 1982.

[28] Borgatti SP, Mehra A, Brass DJ, Labianca G (2009). Network analysis in the social sciences. Sci.

[29] Feld SL (1981). The focused organization of social ties. Am J Sociol.

[30] Valente T (2010). Social networks and health models, methods, and applications.

[31] Cohen S, Wills TA (1985). Stress, social support, and the buffering hypothesis. Psychol Bull.

[32] Cialdini R, Kallgren C, Reno R (1991). A focus theory of normative conduct: A theoretical refinement and reevaluation of the role of norms in human behavior. Adv Exp Soc Psychol.

[33] Dalstrom M (2020). Medicaid, motherhood, and the challenges of having a healthy pregnancy amidst changing social networks. Women Birth : J Austr Coll Midwives.

[34] Lapinski MK, Anderson J, Cruz S, Lapine P (2015). Social networks and the communication of norms about prenatal care in rural Mexico. J Health Commun.

[35] Stokes E, Dumbaya I, Owens S, Brabin L (2008). The right to remain silent: a qualitative study of the medical and social ramifications of pregnancy disclosure for Gambian women BJOG:. Int J of Obstet Gynaecol.

[36] Granovetter MS (1973). The strength of weak ties. Am J Sociol.

[37] Boulay M, Valente T (1999). The relationship of social affiliation and interpersonal discussion to family planning knowledge, attitudes and practice. Int Fam Plan Perspect.

[38] Small M (2017). Someone to talk to.

[39] Rubin H, Rubin I (2005). Qualitative interviewing the art of hearing data.

[40] Braun V, Clarke V. Thematic analysis. In: Cooper H, Camic PM, Long DL, Panter AT, Rindskopf D, Sher KJ, editors. APA handbook of research methods in psychology, Vol. 2. Research designs: Quantitative, qualitative, neuropsychological, and biological (pp. 57–71). American Psychological Association.

[41] Smith JA, Osborn M. Interpretative Phenomenological Analysis. In: Breakwell GM, editor. Doing Social Psychology Research. England: Blackwell Publishing; 2004. p. 229–54.

[42] Simkhada B, Porter MA, van Teijlingen ERT. The role of mothers-in-law in antenatal care decision-making in Nepal: a qualitative study. BMC Pregnancy Childbirth. 2010;10(34):1–10. 10.1186/1471-2393-10-34 PMC2910658 20594340

[43] Comfort AB, Harper C, Tsai A, Perkins J, Moody J, Rasolofomana JR (2021). The association between men's family planning networks and contraceptive use among their female partners: an egocentric network study in Madagascar. BMC Public Health.

[44] Comfort AB, Harper CC, Tsai AC, Moody J, Perkins JM, Rasolofomana JR (2021). Social and provider networks and women's contraceptive use: Evidence from Madagascar. Contracept.

